# Crystal structure of a samarium(III) nitrate chain cross-linked by a bis-carbamoyl­methyl­phosphine oxide ligand

**DOI:** 10.1107/S1600536814020078

**Published:** 2014-09-13

**Authors:** Julie A. Stoscup, Richard J. Staples, Shannon M. Biros

**Affiliations:** a Department of Chemistry, Grand Valley State University, Allendale, MI 49401, USA; b Center for Crystallographic Research, Department of Chemistry, Michigan State University, East Lansing, MI 48824, USA

**Keywords:** crystal structure, carbamoyl­methyl­phosphine oxide (CMPO), rare earth element, metal–organic polymer

## Abstract

In the title compound poly[aqua­bis­(μ-nitrato-κ^4^ *O*,*O*′:*O*,*O*′′)tetra­kis­(nitrato-κ^2^ *O*,*O*′){μ_4_-tetra­ethyl [(ethane-1,2-diyl)bis(aza­nedi­yl)bis­(2-oxo­ethane-2,1-di­yl)]di­phospho­nate-κ^2^ *O*,*O*′}disamarium(III)], [Sm_2_(NO_3_)_6_(C_14_H_30_N_2_O_8_P_2_)(H_2_O)]_*n*_, a 12-coordinate Sm^III^ and a nine-coordinate Sm^III^ cation are alternately linked *via* shared bis-bidentate nitrate anions into a corrugated chain extending parallel to the *a* axis. The nine-coordinate Sm^III^ atom of this chain is also chelated by a bidentate, yet flexible, carbamoyl­methyl­phoshine oxide (CMPO) ligand and bears one water mol­ecule. This water mol­ecule is hydrogen bonded to nitrate groups bonded to the 12-coordinate Sm^III^ cation. The CMPO ligand, which lies about an inversion center, links neighboring chains along the *c* axis, forming sheets parallel to the *ac* plane. Hydrogen bonds between the amide NH group and metal-bound nitrate anions are also present in these sheets. The sheets are packed along the *b* axis through only van der Waals inter­actions.

## Abstract 2

The crystal structure of the title compound consists of a polymeric chain of Sm^III^ cations and nitrate anions, cross-linked in two dimensions with an organic ligand.

## Chemical context   

The carbamoyl­methyl­phosphine oxide (CMPO) moiety has been well studied as a chelating group for lanthanides and actinides. To this end, this bidentate phosphor­yl/carbonyl moiety is a component of the TRUEX process for the treatment of nuclear waste (Siddall, 1963 ▶; Horwitz *et al.*, 1985 ▶). A handful of ligands bearing CMPO groups linked through tri- and tetra­podal caps have been reported in the literature in an attempt to increase the binding strength and selectivity toward *f*-elements (Arnaud-Neu *et al.*, 1996 ▶; Peters *et al.*, 2002 ▶; Sharova *et al.*, 2012 ▶; Sartain *et al.*, 2014 ▶). The title compound, [Sm_2_(NO_3_)_6_(C_14_H_30_N_2_O_8_P_2_)(H_2_O)], is another representative.
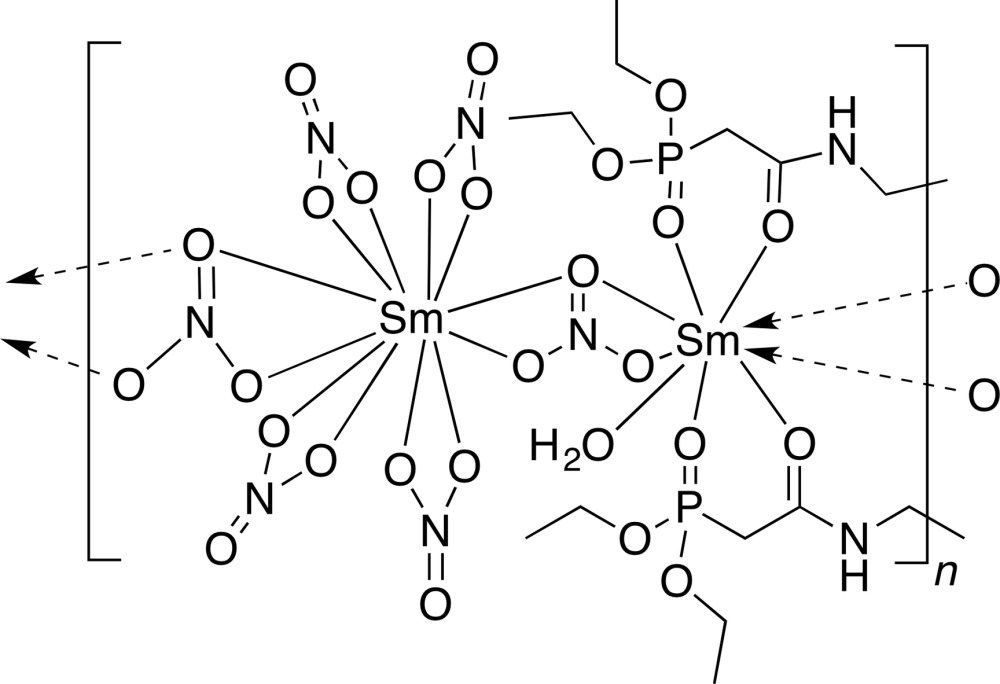


## Structural commentary   

**Figure 1:**
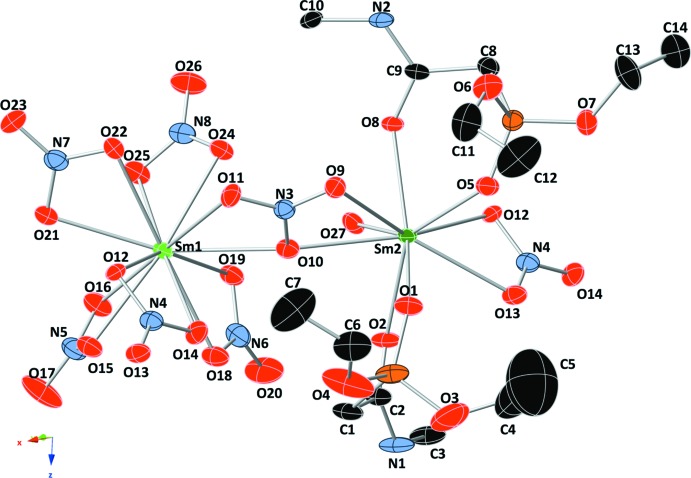
The coordination environments of the Sm^III^ cations of the title compound, showing displacement ellipsoids at the 50% probability level and the atom-numbering scheme. H atoms have been omitted for clarity.

The asymmetric unit of the title compound contains two Sm^III^ ions, one nine-coordinate and one 12-coordinate, two halves of the di-CMPO ligand tetra­ethyl [(ethane-1,2-diyl)bis(aza­nedi­yl)bis­(2-oxo­ethane-2,1-di­yl)]di­phospho­nate, six nitrate anions and one coordinating water mol­ecule (Fig. 1 ▶). The 12-coord­inate Sm^III^ cation (Sm1) is surrounded by six bidentate nitrate ions [range of Sm—O bond lengths = 2.485 (3)–2.705 (3) Å], while the nine-coordinate Sm^III^ cation bears another bidentate nitrate ligand, one water mol­ecule, and two CMPO groups from separate organic ligands [range of Sm—O bond lengths = 2.340 (3)–2.625 (3) Å].

The large displacement parameters of the methyl group (C5) are likely due to large thermal motion of this terminal group (see *Refinement* section for more discussion on the treatment of this disorder).

**Figure 2:**
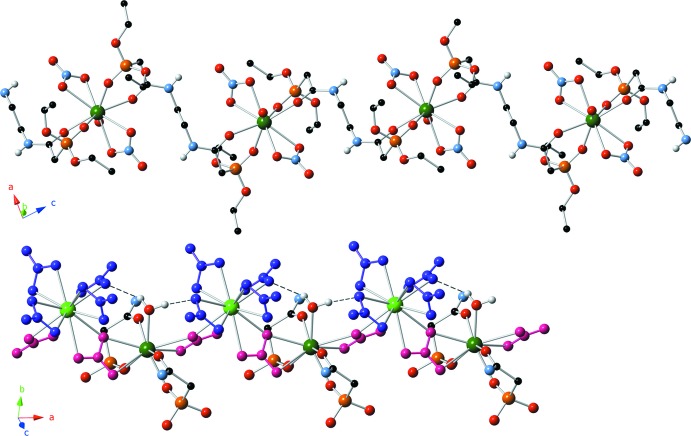
(Top) The Sm2 cations of each metal chain are linked to a neighboring metal chain *via* the di-CMPO organic ligands. Color codes: black C, light green Sm1, dark green Sm2, red O, blue N, and orange P. (Bottom) The metal chain showing alternating Sm1 and Sm2 cations, linked through bridging bis-bidentate nitrate groups shown in pink. Non-bridging nitrate groups are shown in purple. Hydrogen bonds between the water mol­ecule on Sm2 and nitrate groups on Sm1 are shown as dashed lines.

**Figure 3:**
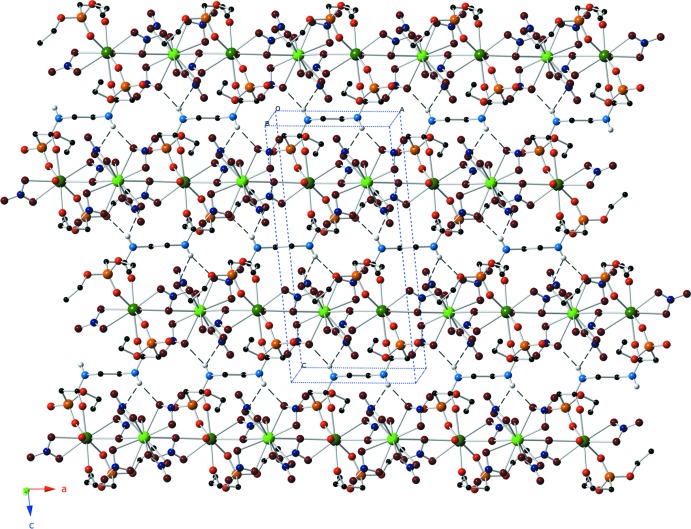
Sheets formed by the cross-linking of the Sm^III^ chains with the di-CMPO organic ligands (viewed down the *b* axis). Hydrogen bonds between the amide NH groups and metal bound nitrate anions are shown as dashed lines.

The Sm^III^ metal cations are bridged through shared bis-bidentate nitrate anions (N3 and N4), forming a corrugated chain (Fig. 2 ▶, bottom) parallel to the *a* axis. In this figure, bridging bis-bidentate nitrate ions are shown in pink, while nitrate ions bound only to the 12-coordinate Sm^III^ cation are shown in purple. The nine-coordinate Sm^III^ ions of the metal chain are also linked by the organic ligand. The organic ligand lies on an inversion center, lies along the *c* axis, and cross-links the metal chains (Fig. 2 ▶, top). This cross-linking results in sheets that extend parallel to the *ac* plane (Fig. 3 ▶).

## Supra­molecular features   

The lanthanide–organic polymer is reinforced through two separate hydrogen-bonding motifs (Table 1 ▶). In the corrugated chain, each H atom (H27*A* and H27*B*) of the water mol­ecule bound to Sm2 forms a hydrogen bond with an O atom of a nitrate group on Sm1 (Fig. 2 ▶, bottom). In the formation of the cross-linked sheets, the amide NH groups (H1 and H2) form hydrogen bonds with O atoms of two separate nitrate groups bound to Sm1 (Fig. 3 ▶). These inter­actions likely act to rigidify both the Sm^III^ chain and the cross-linked organometallic sheets.

**Figure 4:**
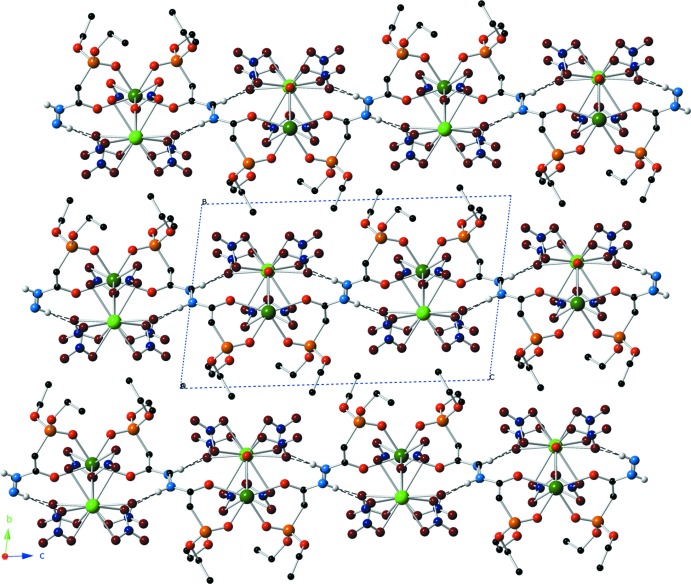
Stacking diagram for the title compound. The horizontal sheets pack vertically with only van der Waals forces.

These metal–organic sheets are stacked along the *b* axis using only van der Waals forces (Fig. 4 ▶). No inter­molecular hydrogen bonds or shared chelating groups are found between the sheets in this third dimension.

## Database survey   

While numerous polymeric structures of lanthanide–organic compounds can be found in the Cambridge Structural Database (CSD; Version 5.35, last update February 2014; Allen, 2002 ▶), one inter­esting feature of this structure is the bidentate bridging of two lanthanides by one shared nitrate group (Fig. 2 ▶, bottom; pink-coloured nitrate groups). At present, only four other examples (Albrecht *et al.*, 2005 ▶; Hashimoto *et al.*, 2000 ▶) with this bidentate bridging motif have been deposited with the CSD.

## Synthesis and crystallization   

The CMPO ligand was prepared following a reported procedure (Hamadouchi *et al.*, 1999 ▶), using ethyl­enedi­amine in place of methyl­amine. This compound was isolated as a white solid. The title metal–ligand coordination polymer was prepared by dissolving the ligand in a minimum amount of aceto­nitrile. To this solution were added 2 molar equivalents of samarium(III) nitrate hexa­hydrate as a solution in aceto­nitrile. The mixture was stirred at room temperature overnight and concentrated under reduced pressure to give an off-white solid. Crystals suitable for X-ray diffraction were grown from vapor diffusion of toluene into a solution of the 2:1 Sm^III^–ligand complex in aceto­nitrile.

## Refinement   

Crystal data, data collection and structure refinement details are summarized in Table 2 ▶. H atoms were placed in calculated positions and constrained to ride on their parent atoms, with *U*_iso_(H) = 1.2*U*_eq_(C,N) for methyl­ene and amino groups, and 1.5*U*_eq_(C,O) for methyl and water groups. C—H distances were restrained to 0.98 Å for methyl and 0.99 Å for methyl­ene H atoms, N—H distances to 0.88 Å and O—H distances to 0.89 Å. One of the methyl groups on the organic ligand (C5) has relatively large displacement ellipsoids that we attribute to large thermal motion of this terminal group. Attempts to model this disorder by assigning two atom locations for C5 or the entire eth­oxy group were unsuccessful. The O3—C4 and C4—C5 bond lengths were constrained using DFIX instructions in *SHELXL* (Sheldrick, 2008 ▶) at 1.46 and 1.54 Å, respectively, to model more accurate bond lengths. The displacement parameters of all methyl groups (C5, C7, C12 and C14) were also treated with ISOR instructions to produce more uniform ellipsoids for these terminal atoms.

## Supplementary Material

Crystal structure: contains datablock(s) I, I_rev1. DOI: 10.1107/S1600536814020078/wm5049sup1.cif

Structure factors: contains datablock(s) I. DOI: 10.1107/S1600536814020078/wm5049Isup2.hkl

CCDC reference: 1023116

Additional supporting information:  crystallographic information; 3D view; checkCIF report

## Figures and Tables

**Table 1:** Hydrogen-bond geometry (Å, °)

*D*—H⋯*A*	*D*—H	H⋯*A*	*D*⋯*A*	*D*—H⋯*A*
O27—H27*A*⋯O21	0.89	2.12	2.772 (4)	130
O27—H27*B*⋯O19^i^	0.89	1.93	2.755 (4)	153
N1—H1⋯O15^ii^	0.88	2.53	3.176 (4)	131
N1—H1⋯O18^ii^	0.88	2.34	3.176 (4)	159
N2—H2⋯O22^iii^	0.88	2.31	3.164 (4)	161
N2—H2⋯O24^iii^	0.88	2.56	3.186 (4)	129

Symmetry codes: (i) 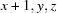; (ii) 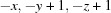; (iii) 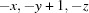.

**Table 2:** Experimental details

Crystal data	
Chemical formula	[Sm_2_(NO_3_)_6_(C_14_H_30_N_2_O_8_P_2_)(H_2_O)]
*M*_r_	1107.11
Crystal system, space group	Triclinic, *P* 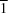
Temperature (K)	173
*a*, *b*, *c* (Å)	8.9416 (7), 11.0128 (9), 18.4635 (15)
α, β, γ (°)	81.441 (1), 83.428 (1), 86.977 (1)
*V* (Å^3^)	1784.9 (2)
*Z*	2
Radiation type	Mo *K*α
μ (mm^−1^)	3.46
Crystal size (mm)	0.21 × 0.20 × 0.07

Data collection	
Diffractometer	Bruker APEXII CCD
Absorption correction	Multi-scan (*SADABS*; Bruker, 2012 ▶)
*T*_min_, *T*_max_	0.648, 0.745
No. of measured, independent and observed [*I* > 2σ(*I*)] reflections	29697, 6597, 5801
*R*_int_	0.034
(sin θ/λ)_max_ (Å^−1^)	0.605

Refinement	
*R*[*F* ^2^ > 2σ(*F* ^2^)], *wR*(*F* ^2^), *S*	0.027, 0.068, 1.09
No. of reflections	6597
No. of parameters	483
No. of restraints	26
H-atom treatment	H-atom parameters constrained
Δρ_max_, Δρ_min_ (e Å^−3^)	1.12, −0.83

Computer programs: *APEX2* and *SAINT* (Bruker, 2012 ▶), *SHELXS97* and *SHELXL97* (Sheldrick, 2008 ▶) and *OLEX2* (Dolomanov *et al.*, 2009 ▶).

## supplementary crystallographic information

### Crystal data

**Table:**

[Sm_2_(NO_3_)_6_(C_14_H_30_N_2_O_8_P_2_)(H_2_O)]	*Z* = 2
*M**_r_* = 1107.11	*F*(000) = 1084
Triclinic, *P*1	*D*_x_ = 2.060 Mg m^−^^3^
*a* = 8.9416 (7) Å	Mo *K*α radiation, λ = 0.71073 Å
*b* = 11.0128 (9) Å	Cell parameters from 9076 reflections
*c* = 18.4635 (15) Å	θ = 2.2–25.5°
α = 81.441 (1)°	µ = 3.46 mm^−^^1^
β = 83.428 (1)°	*T* = 173 K
γ = 86.977 (1)°	Plate, colourless
*V* = 1784.9 (2) Å^3^	0.21 × 0.20 × 0.07 mm

### Data collection

**Table:**

Bruker APEXII CCD diffractometer	5801 reflections with *I* > 2σ(*I*)
Graphite monochromator	*R*_int_ = 0.034
φ and ω scans	θ_max_ = 25.5°, θ_min_ = 1.9°
Absorption correction: multi-scan (*SADABS*; Bruker, 2012)	*h* = −10→10
*T*_min_ = 0.648, *T*_max_ = 0.745	*k* = −13→13
29697 measured reflections	*l* = −22→22
6597 independent reflections	

### Refinement

**Table:**

Refinement on *F*^2^	Primary atom site location: structure-invariant direct methods
Least-squares matrix: full	Hydrogen site location: mixed
*R*[*F*^2^ > 2σ(*F*^2^)] = 0.027	H-atom parameters constrained
*wR*(*F*^2^) = 0.068	*w* = 1/[σ^2^(*F*_o_^2^) + (0.031*P*)^2^ + 3.3158*P*] where *P* = (*F*_o_^2^ + 2*F*_c_^2^)/3
*S* = 1.09	(Δ/σ)_max_ = 0.001
6597 reflections	Δρ_max_ = 1.12 e Å^−^^3^
483 parameters	Δρ_min_ = −0.83 e Å^−^^3^
26 restraints	

### Special details

**Table:**

Geometry. All e.s.d.'s (except the e.s.d. in the dihedral angle between two l.s. planes) are estimated using the full covariance matrix. The cell e.s.d.'s are taken into account individually in the estimation of e.s.d.'s in distances, angles and torsion angles; correlations between e.s.d.'s in cell parameters are only used when they are defined by crystal symmetry. An approximate (isotropic) treatment of cell e.s.d.'s is used for estimating e.s.d.'s involving l.s. planes.

### Fractional atomic coordinates and isotropic or equivalent isotropic displacement parameters (Å^2^)

**Table:**

	*x*	*y*	*z*	*U*_iso_*/*U*_eq_	
C1	0.4236 (5)	0.3466 (4)	0.4463 (2)	0.0310 (11)	
H1A	0.4364	0.3241	0.4992	0.037*	
H1B	0.5133	0.3912	0.4219	0.037*	
C2	0.2843 (5)	0.4287 (4)	0.4379 (2)	0.0251 (9)	
C3	0.0696 (5)	0.5385 (4)	0.4953 (2)	0.0344 (11)	
H3A	0.0736	0.5935	0.4476	0.041*	
H3B	0.0629	0.5904	0.5350	0.041*	
C6	0.6269 (6)	0.0574 (5)	0.3611 (3)	0.0445 (13)	
H6A	0.6609	−0.0172	0.3928	0.053*	
H6B	0.5479	0.0343	0.3327	0.053*	
C7	0.7535 (9)	0.1088 (6)	0.3109 (4)	0.078 (2)	
H7A	0.7214	0.1874	0.2837	0.117*	
H7B	0.7881	0.0516	0.2761	0.117*	
H7C	0.8360	0.1220	0.3392	0.117*	
C8	0.0825 (5)	0.3555 (4)	0.0759 (2)	0.0220 (9)	
H8A	0.0697	0.3371	0.0262	0.026*	
H8B	−0.0102	0.3995	0.0945	0.026*	
C9	0.2156 (5)	0.4353 (4)	0.0718 (2)	0.0195 (8)	
C10	0.4285 (5)	0.5407 (4)	−0.0025 (2)	0.0254 (9)	
H10A	0.4205	0.5951	0.0361	0.030*	
H10B	0.4340	0.5932	−0.0511	0.030*	
C11	0.3455 (6)	0.0601 (5)	0.1246 (3)	0.0476 (14)	
H11A	0.3993	0.1039	0.1563	0.057*	
H11B	0.4202	0.0350	0.0853	0.057*	
C12	0.2811 (8)	−0.0504 (6)	0.1690 (4)	0.070 (2)	
H12A	0.2300	−0.0957	0.1378	0.104*	
H12B	0.2085	−0.0266	0.2088	0.104*	
H12C	0.3619	−0.1029	0.1902	0.104*	
C13	−0.0892 (6)	0.0737 (5)	0.0967 (3)	0.0457 (13)	
H13A	−0.0879	−0.0146	0.1173	0.055*	
H13B	−0.0212	0.0841	0.0502	0.055*	
C14	−0.2433 (6)	0.1154 (6)	0.0820 (3)	0.0557 (15)	
H14A	−0.2797	0.0649	0.0486	0.084*	
H14B	−0.2432	0.2016	0.0590	0.084*	
H14C	−0.3096	0.1075	0.1284	0.084*	
N1	0.2069 (4)	0.4607 (4)	0.49749 (18)	0.0324 (9)	
H1	0.2392	0.4342	0.5405	0.039*	
N2	0.2952 (4)	0.4660 (3)	0.00718 (17)	0.0238 (8)	
H2	0.2666	0.4402	−0.0318	0.029*	
N3	0.5773 (4)	0.4028 (3)	0.20518 (17)	0.0187 (7)	
N4	−0.0810 (4)	0.4035 (3)	0.30951 (18)	0.0201 (7)	
N5	−0.1376 (4)	0.7647 (3)	0.34168 (19)	0.0282 (8)	
N6	−0.5159 (4)	0.6838 (3)	0.3522 (2)	0.0276 (8)	
N7	−0.0156 (4)	0.6943 (3)	0.11928 (19)	0.0244 (8)	
N8	−0.4015 (4)	0.7661 (3)	0.11347 (19)	0.0246 (8)	
O1	0.3540 (3)	0.2348 (3)	0.33183 (15)	0.0264 (7)	
O2	0.2458 (3)	0.4644 (3)	0.37532 (14)	0.0256 (7)	
O4	0.5669 (5)	0.1506 (4)	0.4063 (2)	0.0675 (14)	
O5	0.1545 (3)	0.2391 (3)	0.20820 (15)	0.0230 (6)	
O6	0.2338 (3)	0.1437 (3)	0.09119 (16)	0.0318 (7)	
O7	−0.0363 (3)	0.1451 (3)	0.14912 (17)	0.0341 (8)	
O8	0.2492 (3)	0.4708 (3)	0.12877 (14)	0.0232 (6)	
O9	0.4987 (3)	0.3265 (3)	0.18599 (15)	0.0224 (6)	
O10	0.5160 (3)	0.4694 (2)	0.25338 (14)	0.0202 (6)	
O11	0.7097 (3)	0.4201 (3)	0.18052 (15)	0.0236 (6)	
O12	−0.0270 (3)	0.4703 (2)	0.24983 (13)	0.0188 (6)	
O13	0.0052 (3)	0.3276 (3)	0.34151 (15)	0.0239 (6)	
O14	−0.2148 (3)	0.4198 (3)	0.33178 (15)	0.0243 (6)	
O15	−0.0976 (3)	0.6526 (3)	0.33775 (15)	0.0259 (7)	
O16	−0.2292 (3)	0.8130 (3)	0.29682 (16)	0.0290 (7)	
O17	−0.0955 (5)	0.8200 (4)	0.3872 (2)	0.0570 (11)	
O18	−0.4056 (3)	0.6115 (3)	0.36950 (15)	0.0266 (7)	
O19	−0.5180 (3)	0.7205 (3)	0.28354 (15)	0.0244 (6)	
O20	−0.6118 (4)	0.7143 (4)	0.39827 (18)	0.0490 (10)	
O21	−0.0206 (3)	0.7294 (3)	0.18220 (15)	0.0245 (6)	
O22	−0.1142 (3)	0.6189 (3)	0.11303 (15)	0.0236 (6)	
O23	0.0773 (4)	0.7313 (3)	0.06865 (17)	0.0422 (9)	
O24	−0.4267 (3)	0.6527 (3)	0.13518 (15)	0.0236 (6)	
O25	−0.3199 (3)	0.8163 (3)	0.15244 (16)	0.0283 (7)	
O26	−0.4522 (4)	0.8224 (3)	0.05930 (17)	0.0397 (8)	
O27	0.2340 (3)	0.6220 (2)	0.24155 (15)	0.0240 (6)	
H27A	0.1968	0.6525	0.1996	0.036*	
H27B	0.3253	0.6510	0.2404	0.036*	
P1	0.40754 (14)	0.21020 (11)	0.40584 (6)	0.0292 (3)	
P2	0.11133 (12)	0.21522 (10)	0.13643 (6)	0.0203 (2)	
Sm1	−0.26393 (2)	0.62766 (2)	0.23940 (2)	0.01687 (7)	
Sm2	0.24755 (2)	0.40037 (2)	0.25726 (2)	0.01562 (7)	
O3	0.3060 (5)	0.1299 (4)	0.4626 (2)	0.0724 (14)	
C4	0.1675 (5)	0.0766 (4)	0.4490 (4)	0.103 (3)	
H4A	0.1224	0.1261	0.4071	0.124*	
H4B	0.0928	0.0707	0.4932	0.124*	
C5	0.2205 (12)	−0.0518 (5)	0.4307 (6)	0.154 (4)	
H5A	0.2050	−0.0574	0.3796	0.231*	
H5B	0.1625	−0.1145	0.4640	0.231*	
H5C	0.3278	−0.0654	0.4370	0.231*	

### Atomic displacement parameters (Å^2^)

**Table:**

	*U*^11^	*U*^22^	*U*^33^	*U*^12^	*U*^13^	*U*^23^
C1	0.029 (2)	0.050 (3)	0.015 (2)	0.011 (2)	−0.0059 (18)	−0.010 (2)
C2	0.030 (2)	0.030 (2)	0.015 (2)	0.0021 (19)	−0.0032 (17)	−0.0046 (17)
C3	0.044 (3)	0.041 (3)	0.016 (2)	0.019 (2)	0.001 (2)	−0.008 (2)
C6	0.046 (3)	0.044 (3)	0.041 (3)	0.024 (3)	−0.002 (2)	−0.012 (2)
C7	0.100 (5)	0.046 (4)	0.078 (5)	0.003 (4)	0.026 (4)	−0.006 (3)
C8	0.023 (2)	0.025 (2)	0.019 (2)	−0.0002 (17)	−0.0043 (17)	−0.0055 (17)
C9	0.025 (2)	0.021 (2)	0.0132 (19)	0.0059 (17)	−0.0054 (16)	−0.0040 (16)
C10	0.034 (2)	0.028 (2)	0.013 (2)	−0.0099 (19)	0.0018 (17)	−0.0010 (17)
C11	0.033 (3)	0.039 (3)	0.067 (4)	0.008 (2)	0.005 (3)	−0.010 (3)
C12	0.066 (4)	0.053 (4)	0.076 (4)	0.026 (3)	0.011 (3)	0.011 (3)
C13	0.046 (3)	0.039 (3)	0.060 (4)	−0.012 (2)	−0.015 (3)	−0.020 (3)
C14	0.043 (3)	0.071 (4)	0.055 (3)	−0.011 (3)	−0.008 (3)	−0.008 (3)
N1	0.041 (2)	0.045 (2)	0.0100 (17)	0.0155 (19)	−0.0028 (16)	−0.0058 (16)
N2	0.032 (2)	0.030 (2)	0.0097 (16)	−0.0050 (16)	−0.0008 (14)	−0.0045 (14)
N3	0.0178 (18)	0.0212 (18)	0.0169 (17)	0.0018 (14)	−0.0026 (14)	−0.0020 (14)
N4	0.0194 (18)	0.0243 (18)	0.0172 (17)	0.0025 (15)	−0.0016 (14)	−0.0067 (14)
N5	0.029 (2)	0.035 (2)	0.0237 (19)	−0.0001 (17)	−0.0069 (16)	−0.0118 (17)
N6	0.0212 (19)	0.038 (2)	0.026 (2)	0.0045 (17)	−0.0037 (16)	−0.0139 (17)
N7	0.0236 (19)	0.029 (2)	0.0202 (19)	−0.0007 (16)	−0.0075 (15)	0.0003 (15)
N8	0.0249 (19)	0.030 (2)	0.0197 (18)	0.0061 (16)	−0.0047 (15)	−0.0061 (16)
O1	0.0335 (17)	0.0276 (16)	0.0181 (15)	0.0058 (13)	−0.0078 (13)	−0.0013 (12)
O2	0.0300 (17)	0.0353 (17)	0.0114 (14)	0.0091 (13)	−0.0024 (12)	−0.0063 (12)
O4	0.066 (3)	0.096 (3)	0.048 (2)	0.059 (3)	−0.030 (2)	−0.039 (2)
O5	0.0294 (16)	0.0226 (15)	0.0176 (14)	−0.0034 (12)	−0.0047 (12)	−0.0020 (12)
O6	0.0329 (18)	0.0307 (17)	0.0309 (17)	0.0097 (14)	0.0035 (14)	−0.0103 (14)
O7	0.0303 (18)	0.045 (2)	0.0297 (17)	−0.0168 (15)	0.0025 (14)	−0.0122 (15)
O8	0.0317 (16)	0.0278 (16)	0.0111 (14)	−0.0085 (13)	−0.0030 (12)	−0.0034 (11)
O9	0.0246 (15)	0.0231 (15)	0.0209 (15)	−0.0056 (12)	0.0008 (12)	−0.0085 (12)
O10	0.0189 (15)	0.0242 (15)	0.0184 (14)	0.0027 (12)	−0.0008 (11)	−0.0081 (12)
O11	0.0175 (15)	0.0278 (16)	0.0243 (15)	−0.0019 (12)	0.0047 (12)	−0.0046 (12)
O12	0.0188 (14)	0.0233 (15)	0.0127 (14)	−0.0006 (11)	0.0015 (11)	0.0002 (11)
O13	0.0219 (15)	0.0302 (16)	0.0172 (14)	0.0083 (13)	−0.0031 (12)	0.0021 (12)
O14	0.0167 (15)	0.0295 (16)	0.0247 (15)	0.0017 (12)	0.0029 (12)	−0.0025 (12)
O15	0.0267 (16)	0.0304 (17)	0.0229 (15)	0.0063 (13)	−0.0092 (13)	−0.0095 (13)
O16	0.0352 (18)	0.0252 (16)	0.0301 (17)	0.0054 (13)	−0.0136 (14)	−0.0095 (13)
O17	0.073 (3)	0.053 (2)	0.059 (3)	0.011 (2)	−0.039 (2)	−0.037 (2)
O18	0.0227 (16)	0.0375 (18)	0.0201 (15)	0.0086 (13)	−0.0052 (12)	−0.0070 (13)
O19	0.0227 (15)	0.0311 (17)	0.0211 (15)	0.0054 (13)	−0.0069 (12)	−0.0083 (13)
O20	0.038 (2)	0.079 (3)	0.0309 (19)	0.0201 (19)	0.0046 (16)	−0.0229 (19)
O21	0.0263 (16)	0.0304 (16)	0.0186 (15)	−0.0029 (13)	−0.0064 (12)	−0.0060 (12)
O22	0.0221 (15)	0.0279 (16)	0.0224 (15)	−0.0047 (13)	−0.0058 (12)	−0.0049 (12)
O23	0.042 (2)	0.061 (2)	0.0218 (17)	−0.0213 (18)	0.0046 (15)	0.0022 (16)
O24	0.0274 (16)	0.0247 (16)	0.0206 (15)	−0.0006 (13)	−0.0091 (12)	−0.0041 (12)
O25	0.0353 (18)	0.0246 (16)	0.0274 (16)	0.0007 (13)	−0.0121 (14)	−0.0049 (13)
O26	0.053 (2)	0.039 (2)	0.0269 (18)	0.0116 (17)	−0.0174 (16)	0.0009 (15)
O27	0.0232 (16)	0.0266 (16)	0.0242 (16)	0.0004 (13)	−0.0107 (13)	−0.0038 (12)
P1	0.0352 (7)	0.0342 (7)	0.0157 (5)	0.0136 (5)	−0.0028 (5)	−0.0003 (5)
P2	0.0215 (6)	0.0216 (5)	0.0182 (5)	−0.0023 (4)	0.0007 (4)	−0.0058 (4)
Sm1	0.01713 (11)	0.02063 (12)	0.01388 (11)	0.00136 (8)	−0.00375 (8)	−0.00494 (8)
Sm2	0.01417 (11)	0.02308 (12)	0.00966 (10)	0.00120 (8)	−0.00118 (7)	−0.00321 (8)
O3	0.111 (4)	0.068 (3)	0.033 (2)	−0.040 (3)	0.005 (2)	0.012 (2)
C4	0.101 (7)	0.116 (7)	0.080 (6)	−0.024 (6)	0.022 (5)	0.011 (5)
C5	0.131 (7)	0.134 (7)	0.206 (9)	−0.005 (6)	−0.006 (7)	−0.064 (7)

### Geometric parameters (Å, º)

**Table:**

C1—H1A	0.9900	N5—O17	1.212 (5)
C1—H1B	0.9900	N5—Sm1	2.939 (3)
C1—C2	1.508 (6)	N6—O18	1.274 (4)
C1—P1	1.794 (5)	N6—O19	1.274 (4)
C2—N1	1.315 (5)	N6—O20	1.207 (4)
C2—O2	1.245 (5)	N6—Sm1	2.993 (3)
C3—C3^i^	1.522 (10)	N7—O21	1.273 (4)
C3—H3A	0.9900	N7—O22	1.268 (4)
C3—H3B	0.9900	N7—O23	1.214 (4)
C3—N1	1.460 (6)	N7—Sm1	2.991 (4)
C6—H6A	0.9900	N8—O24	1.277 (4)
C6—H6B	0.9900	N8—O25	1.279 (4)
C6—C7	1.461 (8)	N8—O26	1.216 (4)
C6—O4	1.461 (6)	N8—Sm1	2.940 (3)
C7—H7A	0.9800	O1—P1	1.482 (3)
C7—H7B	0.9800	O1—Sm2	2.344 (3)
C7—H7C	0.9800	O2—Sm2	2.387 (3)
C8—H8A	0.9900	O4—P1	1.537 (4)
C8—H8B	0.9900	O5—P2	1.485 (3)
C8—C9	1.504 (6)	O5—Sm2	2.340 (3)
C8—P2	1.792 (4)	O6—P2	1.553 (3)
C9—N2	1.325 (5)	O7—P2	1.542 (3)
C9—O8	1.249 (4)	O8—Sm2	2.382 (3)
C10—C10^ii^	1.526 (9)	O9—Sm2	2.625 (3)
C10—H10A	0.9900	O10—Sm1^iii^	2.663 (3)
C10—H10B	0.9900	O10—Sm2	2.546 (3)
C10—N2	1.463 (5)	O11—Sm1^iii^	2.705 (3)
C11—H11A	0.9900	O12—Sm1	2.671 (3)
C11—H11B	0.9900	O12—Sm2	2.547 (3)
C11—C12	1.467 (8)	O13—Sm2	2.607 (3)
C11—O6	1.449 (6)	O14—Sm1	2.692 (3)
C12—H12A	0.9800	O15—Sm1	2.528 (3)
C12—H12B	0.9800	O16—Sm1	2.485 (3)
C12—H12C	0.9800	O18—Sm1	2.570 (3)
C13—H13A	0.9900	O19—Sm1	2.540 (3)
C13—H13B	0.9900	O21—Sm1	2.546 (3)
C13—C14	1.472 (7)	O22—Sm1	2.566 (3)
C13—O7	1.466 (5)	O24—Sm1	2.518 (3)
C14—H14A	0.9800	O25—Sm1	2.495 (3)
C14—H14B	0.9800	O27—H27A	0.8910
C14—H14C	0.9800	O27—H27B	0.8889
N1—H1	0.8800	O27—Sm2	2.413 (3)
N2—H2	0.8800	P1—O3	1.515 (4)
N3—O9	1.238 (4)	Sm1—O10^iv^	2.663 (3)
N3—O10	1.292 (4)	Sm1—O11^iv^	2.705 (3)
N3—O11	1.233 (4)	O3—C4	1.4609 (2)
N3—Sm2	2.995 (3)	C4—H4A	0.9900
N4—O12	1.290 (4)	C4—H4B	0.9900
N4—O13	1.238 (4)	C4—C5	1.5409 (2)
N4—O14	1.232 (4)	C5—H5A	0.9800
N4—Sm2	2.986 (3)	C5—H5B	0.9800
N5—O15	1.277 (4)	C5—H5C	0.9800
N5—O16	1.272 (4)		
			
H1A—C1—H1B	108.2	O3—P1—O4	106.8 (3)
C2—C1—H1A	109.6	O5—P2—C8	111.44 (18)
C2—C1—H1B	109.6	O5—P2—O6	114.34 (17)
C2—C1—P1	110.1 (3)	O5—P2—O7	109.63 (17)
P1—C1—H1A	109.6	O6—P2—C8	103.35 (18)
P1—C1—H1B	109.6	O7—P2—C8	108.30 (19)
N1—C2—C1	118.6 (4)	O7—P2—O6	109.49 (18)
O2—C2—C1	119.6 (4)	O10^iv^—Sm1—O11^iv^	47.58 (8)
O2—C2—N1	121.8 (4)	O10^iv^—Sm1—O12	99.82 (8)
C3^i^—C3—H3A	109.4	O10^iv^—Sm1—O14	66.48 (8)
C3^i^—C3—H3B	109.4	O12—Sm1—O11^iv^	65.78 (8)
H3A—C3—H3B	108.0	O12—Sm1—O14	47.61 (8)
N1—C3—C3^i^	111.2 (5)	O14—Sm1—O11^iv^	66.19 (9)
N1—C3—H3A	109.4	O15—Sm1—O10^iv^	125.69 (9)
N1—C3—H3B	109.4	O15—Sm1—O11^iv^	126.11 (9)
H6A—C6—H6B	108.4	O15—Sm1—O12	64.28 (8)
C7—C6—H6A	110.1	O15—Sm1—O14	65.88 (9)
C7—C6—H6B	110.1	O15—Sm1—O18	66.49 (9)
C7—C6—O4	108.0 (5)	O15—Sm1—O19	104.37 (9)
O4—C6—H6A	110.1	O15—Sm1—O21	69.29 (9)
O4—C6—H6B	110.1	O15—Sm1—O22	112.67 (9)
C6—C7—H7A	109.5	O16—Sm1—O10^iv^	133.60 (9)
C6—C7—H7B	109.5	O16—Sm1—O11^iv^	177.10 (9)
C6—C7—H7C	109.5	O16—Sm1—O12	111.44 (9)
H7A—C7—H7B	109.5	O16—Sm1—O14	111.46 (9)
H7A—C7—H7C	109.5	O16—Sm1—O15	51.01 (9)
H7B—C7—H7C	109.5	O16—Sm1—O18	69.05 (10)
H8A—C8—H8B	108.2	O16—Sm1—O19	69.79 (10)
C9—C8—H8A	109.7	O16—Sm1—O21	69.86 (10)
C9—C8—H8B	109.7	O16—Sm1—O22	115.60 (10)
C9—C8—P2	109.7 (3)	O16—Sm1—O24	117.28 (9)
P2—C8—H8A	109.7	O16—Sm1—O25	70.34 (9)
P2—C8—H8B	109.7	O18—Sm1—O10^iv^	69.92 (9)
N2—C9—C8	118.9 (3)	O18—Sm1—O11^iv^	110.60 (9)
O8—C9—C8	119.8 (4)	O18—Sm1—O12	105.95 (8)
O8—C9—N2	121.3 (4)	O18—Sm1—O14	63.31 (9)
C10^ii^—C10—H10A	109.5	O19—Sm1—O10^iv^	67.58 (9)
C10^ii^—C10—H10B	109.5	O19—Sm1—O11^iv^	112.32 (9)
H10A—C10—H10B	108.1	O19—Sm1—O12	154.83 (8)
N2—C10—C10^ii^	110.7 (4)	O19—Sm1—O14	107.65 (9)
N2—C10—H10A	109.5	O19—Sm1—O18	49.87 (9)
N2—C10—H10B	109.5	O19—Sm1—O21	130.76 (9)
H11A—C11—H11B	107.7	O19—Sm1—O22	134.81 (9)
C12—C11—H11A	108.9	O21—Sm1—O10^iv^	156.23 (8)
C12—C11—H11B	108.9	O21—Sm1—O11^iv^	109.21 (8)
O6—C11—H11A	108.9	O21—Sm1—O12	68.54 (9)
O6—C11—H11B	108.9	O21—Sm1—O14	112.57 (9)
O6—C11—C12	113.4 (5)	O21—Sm1—O18	132.34 (9)
C11—C12—H12A	109.5	O21—Sm1—O22	49.75 (9)
C11—C12—H12B	109.5	O22—Sm1—O10^iv^	107.16 (8)
C11—C12—H12C	109.5	O22—Sm1—O11^iv^	64.52 (8)
H12A—C12—H12B	109.5	O22—Sm1—O12	68.75 (8)
H12A—C12—H12C	109.5	O22—Sm1—O14	110.52 (9)
H12B—C12—H12C	109.5	O22—Sm1—O18	173.74 (9)
H13A—C13—H13B	108.2	O24—Sm1—O10^iv^	64.44 (9)
C14—C13—H13A	109.7	O24—Sm1—O11^iv^	65.55 (9)
C14—C13—H13B	109.7	O24—Sm1—O12	124.03 (8)
O7—C13—H13A	109.7	O24—Sm1—O14	126.46 (9)
O7—C13—H13B	109.7	O24—Sm1—O15	167.55 (9)
O7—C13—C14	109.9 (4)	O24—Sm1—O18	115.68 (9)
C13—C14—H14A	109.5	O24—Sm1—O19	71.63 (9)
C13—C14—H14B	109.5	O24—Sm1—O21	103.97 (9)
C13—C14—H14C	109.5	O24—Sm1—O22	66.59 (9)
H14A—C14—H14B	109.5	O25—Sm1—O10^iv^	110.95 (9)
H14A—C14—H14C	109.5	O25—Sm1—O11^iv^	112.06 (9)
H14B—C14—H14C	109.5	O25—Sm1—O12	133.87 (9)
C2—N1—C3	122.8 (4)	O25—Sm1—O14	177.43 (9)
C2—N1—H1	118.6	O25—Sm1—O15	116.50 (9)
C3—N1—H1	118.6	O25—Sm1—O18	116.35 (10)
C9—N2—C10	123.2 (3)	O25—Sm1—O19	71.06 (10)
C9—N2—H2	118.4	O25—Sm1—O21	69.65 (10)
C10—N2—H2	118.4	O25—Sm1—O22	69.77 (9)
O9—N3—O10	117.9 (3)	O25—Sm1—O24	51.13 (9)
O9—N3—Sm2	60.84 (18)	N4—Sm2—N3	178.81 (9)
O10—N3—Sm2	57.50 (17)	O1—Sm2—N3	75.33 (10)
O11—N3—O9	124.0 (3)	O1—Sm2—N4	105.41 (10)
O11—N3—O10	118.1 (3)	O1—Sm2—O2	74.17 (10)
O11—N3—Sm2	171.5 (3)	O1—Sm2—O8	136.97 (10)
O12—N4—Sm2	57.91 (17)	O1—Sm2—O9	71.48 (9)
O13—N4—O12	117.9 (3)	O1—Sm2—O10	78.85 (10)
O13—N4—Sm2	60.43 (19)	O1—Sm2—O12	130.32 (9)
O14—N4—O12	118.2 (3)	O1—Sm2—O13	81.06 (9)
O14—N4—O13	123.9 (3)	O1—Sm2—O27	139.51 (9)
O14—N4—Sm2	172.1 (3)	O2—Sm2—N3	101.03 (9)
O15—N5—Sm1	58.92 (18)	O2—Sm2—N4	78.34 (9)
O16—N5—O15	115.8 (3)	O2—Sm2—O9	121.05 (9)
O16—N5—Sm1	56.92 (18)	O2—Sm2—O10	77.94 (9)
O17—N5—O15	122.0 (4)	O2—Sm2—O12	91.77 (9)
O17—N5—O16	122.1 (4)	O2—Sm2—O13	69.90 (10)
O17—N5—Sm1	175.1 (3)	O2—Sm2—O27	71.63 (9)
O18—N6—O19	115.4 (3)	O5—Sm2—N3	105.48 (10)
O18—N6—Sm1	58.56 (19)	O5—Sm2—N4	75.60 (9)
O19—N6—Sm1	57.19 (18)	O5—Sm2—O1	81.18 (10)
O20—N6—O18	121.7 (4)	O5—Sm2—O2	137.54 (10)
O20—N6—O19	122.9 (4)	O5—Sm2—O8	74.70 (9)
O20—N6—Sm1	175.0 (3)	O5—Sm2—O9	81.16 (9)
O21—N7—Sm1	57.56 (19)	O5—Sm2—O10	130.47 (9)
O22—N7—O21	115.6 (3)	O5—Sm2—O12	78.43 (9)
O22—N7—Sm1	58.44 (19)	O5—Sm2—O13	72.55 (9)
O23—N7—O21	121.9 (4)	O5—Sm2—O27	139.29 (9)
O23—N7—O22	122.4 (3)	O8—Sm2—N3	77.51 (9)
O23—N7—Sm1	173.9 (3)	O8—Sm2—N4	102.40 (9)
O24—N8—O25	115.6 (3)	O8—Sm2—O2	144.24 (10)
O24—N8—Sm1	58.38 (18)	O8—Sm2—O9	70.00 (9)
O25—N8—Sm1	57.38 (19)	O8—Sm2—O10	90.33 (9)
O26—N8—O24	121.9 (4)	O8—Sm2—O12	78.92 (9)
O26—N8—O25	122.4 (4)	O8—Sm2—O13	122.96 (9)
O26—N8—Sm1	176.9 (3)	O8—Sm2—O27	72.66 (9)
P1—O1—Sm2	137.71 (18)	O9—Sm2—N3	24.32 (8)
C2—O2—Sm2	141.6 (3)	O9—Sm2—N4	156.74 (9)
C6—O4—P1	124.7 (4)	O10—Sm2—N3	25.35 (8)
P2—O5—Sm2	138.15 (17)	O10—Sm2—N4	153.65 (8)
C11—O6—P2	123.2 (3)	O10—Sm2—O9	49.56 (8)
C13—O7—P2	125.0 (3)	O10—Sm2—O12	145.44 (9)
C9—O8—Sm2	139.8 (3)	O10—Sm2—O13	145.66 (8)
N3—O9—Sm2	94.8 (2)	O12—Sm2—N3	154.01 (8)
N3—O10—Sm1^iii^	96.9 (2)	O12—Sm2—N4	25.41 (8)
N3—O10—Sm2	97.1 (2)	O12—Sm2—O9	146.35 (8)
Sm2—O10—Sm1^iii^	156.84 (11)	O12—Sm2—O13	49.69 (8)
N3—O11—Sm1^iii^	96.5 (2)	O13—Sm2—N3	156.28 (8)
N4—O12—Sm1	96.29 (19)	O13—Sm2—N4	24.38 (8)
N4—O12—Sm2	96.7 (2)	O13—Sm2—O9	144.47 (9)
Sm2—O12—Sm1	157.45 (12)	O27—Sm2—N3	90.54 (9)
N4—O13—Sm2	95.2 (2)	O27—Sm2—N4	88.30 (9)
N4—O14—Sm1	96.8 (2)	O27—Sm2—O9	109.18 (9)
N5—O15—Sm1	95.4 (2)	O27—Sm2—O10	73.42 (9)
N5—O16—Sm1	97.7 (2)	O27—Sm2—O12	72.02 (9)
N6—O18—Sm1	96.4 (2)	O27—Sm2—O13	106.34 (9)
N6—O19—Sm1	97.9 (2)	C4—O3—P1	125.4 (4)
N7—O21—Sm1	97.5 (2)	O3—C4—H4A	111.1
N7—O22—Sm1	96.7 (2)	O3—C4—H4B	111.1
N8—O24—Sm1	96.0 (2)	O3—C4—C5	103.4 (5)
N8—O25—Sm1	97.0 (2)	H4A—C4—H4B	109.0
H27A—O27—H27B	108.3	C5—C4—H4A	111.1
Sm2—O27—H27A	110.9	C5—C4—H4B	111.1
Sm2—O27—H27B	110.2	C4—C5—H5A	109.5
O1—P1—C1	113.43 (19)	C4—C5—H5B	109.5
O1—P1—O4	113.95 (19)	C4—C5—H5C	109.5
O1—P1—O3	114.3 (2)	H5A—C5—H5B	109.5
O4—P1—C1	102.9 (2)	H5A—C5—H5C	109.5
O3—P1—C1	104.4 (2)	H5B—C5—H5C	109.5
			
C1—C2—N1—C3	−179.2 (4)	O11—N3—O10—Sm2	171.6 (3)
C1—C2—O2—Sm2	33.1 (7)	O12—N4—O13—Sm2	7.0 (3)
C1—P1—O3—C4	125.6 (4)	O12—N4—O14—Sm1	−10.4 (3)
C2—C1—P1—O1	46.3 (4)	O13—N4—O12—Sm1	−168.7 (3)
C2—C1—P1—O4	169.9 (3)	O13—N4—O12—Sm2	−7.1 (3)
C2—C1—P1—O3	−78.7 (4)	O13—N4—O14—Sm1	168.7 (3)
C3^i^—C3—N1—C2	92.6 (6)	O14—N4—O12—Sm1	10.5 (3)
C6—O4—P1—C1	−161.4 (5)	O14—N4—O12—Sm2	172.0 (3)
C6—O4—P1—O1	−38.2 (6)	O14—N4—O13—Sm2	−172.2 (3)
C6—O4—P1—O3	88.9 (5)	O15—N5—O16—Sm1	2.9 (4)
C7—C6—O4—P1	116.4 (6)	O16—N5—O15—Sm1	−2.8 (4)
C8—C9—N2—C10	−178.6 (4)	O17—N5—O15—Sm1	174.4 (4)
C8—C9—O8—Sm2	34.7 (6)	O17—N5—O16—Sm1	−174.3 (4)
C9—C8—P2—O5	50.5 (3)	O18—N6—O19—Sm1	−6.4 (4)
C9—C8—P2—O6	−72.7 (3)	O19—N6—O18—Sm1	6.3 (4)
C9—C8—P2—O7	171.2 (3)	O20—N6—O18—Sm1	−174.2 (4)
C10^ii^—C10—N2—C9	93.4 (5)	O20—N6—O19—Sm1	174.1 (4)
C11—O6—P2—C8	148.3 (4)	O21—N7—O22—Sm1	6.8 (3)
C11—O6—P2—O5	27.0 (4)	O22—N7—O21—Sm1	−6.9 (3)
C11—O6—P2—O7	−96.5 (4)	O23—N7—O21—Sm1	172.8 (3)
C12—C11—O6—P2	66.9 (6)	O23—N7—O22—Sm1	−172.9 (4)
C13—O7—P2—C8	77.8 (4)	O24—N8—O25—Sm1	3.8 (3)
C13—O7—P2—O5	−160.4 (4)	O25—N8—O24—Sm1	−3.7 (3)
C13—O7—P2—O6	−34.2 (4)	O26—N8—O24—Sm1	176.4 (3)
C14—C13—O7—P2	−126.2 (4)	O26—N8—O25—Sm1	−176.4 (3)
N1—C2—O2—Sm2	−147.0 (4)	P1—C1—C2—N1	122.9 (4)
N2—C9—O8—Sm2	−145.0 (3)	P1—C1—C2—O2	−57.2 (5)
O1—P1—O3—C4	1.1 (5)	P1—O3—C4—C5	93.5 (7)
O2—C2—N1—C3	0.9 (7)	P2—C8—C9—N2	119.1 (4)
O4—P1—O3—C4	−125.8 (4)	P2—C8—C9—O8	−60.7 (4)
O8—C9—N2—C10	1.2 (6)	Sm2—N3—O10—Sm1^iii^	−161.56 (15)
O9—N3—O10—Sm1^iii^	−169.1 (3)	Sm2—N4—O12—Sm1	−161.52 (15)
O9—N3—O10—Sm2	−7.6 (3)	Sm2—O1—P1—C1	−11.9 (3)
O9—N3—O11—Sm1^iii^	169.2 (3)	Sm2—O1—P1—O4	−129.3 (3)
O10—N3—O9—Sm2	7.3 (3)	Sm2—O1—P1—O3	107.6 (3)
O10—N3—O11—Sm1^iii^	−9.9 (3)	Sm2—O5—P2—C8	−17.6 (3)
O11—N3—O9—Sm2	−171.8 (3)	Sm2—O5—P2—O6	99.2 (3)
O11—N3—O10—Sm1^iii^	10.1 (3)	Sm2—O5—P2—O7	−137.4 (2)

Symmetry codes: (i) −*x*, −*y*+1, −*z*+1; (ii) −*x*+1, −*y*+1, −*z*; (iii) *x*+1, *y*, *z*; (iv) *x*−1, *y*, *z*.

### Hydrogen-bond geometry (Å, º)

**Table:**

*D*—H···*A*	*D*—H	H···*A*	*D*···*A*	*D*—H···*A*
O27—H27*A*···O21	0.89	2.12	2.772 (4)	130
O27—H27*B*···O19^iii^	0.89	1.93	2.755 (4)	153
N1—H1···O15^i^	0.88	2.53	3.176 (4)	131
N1—H1···O18^i^	0.88	2.34	3.176 (4)	159
N2—H2···O22^v^	0.88	2.31	3.164 (4)	161
N2—H2···O24^v^	0.88	2.56	3.186 (4)	129

Symmetry codes: (i) −*x*, −*y*+1, −*z*+1; (iii) *x*+1, *y*, *z*; (v) −*x*, −*y*+1, −*z*.
